# Supplementary material for: Nonlinear Mixed-Effect Pharmacokinetic Modeling and Distribution of Doxycycline in Healthy Female Donkeys after Multiple Intragastric Dosing–Preliminary Investigation
Source: Animals (Basel). 2021 Jul 9;11(7):2047. doi: 10.3390/ani11072047 (PMC8300337; doi:10.3390/ani11072047)
Supplement: Supplementary file 1 [file animals-11-02047-s001.zip › animals-1217840-supplementary.pdf]

Supplemental Table S1: Correlation matrix for donkey serum doxycycline concentration after intragastric administration of doxycycline at 10 mg/kg q12h from 0 to 48 hours in 8 healthy jennies.

	**R.S.E.(%)**								
**K_a_pop**	45.3	1							
**V_z/F_pop**	24.4	0.023098	1						
**K_{el}_pop**	9.82	-0.11099	-0.1526	1					
**Omega_ V_z/F**	27.6	0	0	0	1				
**Gamma_ K_a**	13.3	0	0	0	2.0534e-5	1			
**Gamma_ V_z/F**	13.7	0	0	0	-0.046754	-0.011113	1		
**a**	31.7	0	0	0	-0.00013074	-0.021655	-0.01886	1	
**b**	16	0	0	0	8.8309e-5	-0.044614	-0.018054	-0.66026	1

Key: K_a, absorption rate constant first order; V_z/F, apparent volume of distribution during terminal phase after non-intravenous administration; K_{el}, elimination rate constant from the central compartment, R.S.E.(%): Root Square Error
